# Piperacillin/tazobactam versus carbapenems for 30-day mortality in patients with ESBL-producing Enterobacterales bloodstream infections: a retrospective, multicenter, non-inferiority, cohort study

**DOI:** 10.1007/s15010-025-02496-x

**Published:** 2025-04-16

**Authors:** Thomas Sahlström Månsson, Alice Askemyr, Torgny Sunnerhagen, Johan Tham, Kristian Riesbeck, Lisa Mellhammar

**Affiliations:** 1 Department of Infectious Diseases, Sahlgrenska University Hospital, Gothenburg, Sweden; 2 Department of Infectious Diseases, Skåne University Hospital, Malmö/Lund, Sweden; 3 Clinical Microbiology, Infection Prevention and Control, Lund, Sweden; 4 Department of Clinical Sciences Lund, Division of Infection Medicine, Lund University, Lund, Sweden; 5 Department of Translational Medicine, Division of Infectious Diseases and Clinical Microbiology, Faculty of Medicine, Lund University, Malmö, Sweden; 6 Clinical Microbiology, Office for Medical Services, Region Skåne, Kristianstad, Sweden; 7 Clinical Research Centre, CRC, Plan 11, Jan Waldenströms Gata 35, Malmö, 205 02 Sweden

**Keywords:** 30-day mortality, Antimicrobial resistance, Enterobacterales, Extended-spectrum beta-lactamase, Non-inferiority, Propensity score matching

## Abstract

**Purpose:**

Antimicrobial resistance increases with the use of broad-spectrum antibiotics. Studies evaluating antibiotic stewardship are in high demand. Is piperacillin/tazobactam non-inferior to carbapenems regarding 30-day mortality among patients with bloodstream infections caused by extended-spectrum beta-lactamase-producing Enterobacterales?

**Methods:**

This retrospective, multicenter, non-inferiority, cohort study assessed adult patients with bloodstream infections caused by extended-spectrum beta-lactamase-producing Enterobacterales in southern Sweden from 2013 to 2022. Patients were categorized according to the first therapy they received two consecutive doses of (piperacillin/tazobactam or a carbapenem). The primary outcome was 30-day all-cause mortality, measured from when the positive blood cultures were taken. The absolute risk difference for this outcome was calculated for all patients, and two propensity score matched cohorts (empirical and effective), with two different delta limits (5% and 2%). Secondary outcomes included intensive care unit admission, early clinical response, superinfections, relapsed infection and one-year mortality.

**Results:**

A total of 644 patients were included. In the piperacillin/tazobactam group, 26/309 patients met the primary outcome, compared to 27/335 patients in the carbapenem group. The absolute risk difference (-0.4%) was statistically significant in the propensity score matched empirical cohort [1-sided 97.5% confidence interval]: -∞ to 4.0, *p* = 0.008). Piperacillin/tazobactam was non-inferior to carbapenems for all the secondary outcomes in the same cohort, except for the early clinical response.

**Conclusion:**

Our findings indicate that piperacillin/tazobactam is non-inferior to carbapenems for treating extended-spectrum beta-lactamase-producing Enterobacterales bloodstream infections, with an acceptable 5% increase in 30-day mortality. We suggest that piperacillin/tazobactam should be used more frequently to decrease antimicrobial resistance.

**Supplementary Information:**

The online version contains supplementary material available at https://doi.org/10.1007/s15010-025-02496-x.

## Introduction

In 2019, nearly 5 million deaths worldwide, were associated with bacterial antimicrobial resistance (AMR), with an estimated 33,000 people dying annually from AMR in Europe. The majority of these deaths are caused by third-generation cephalosporin-resistant Enterobacterales [1]. Data from 29 European countries showed that 13.8% of invasive *E. coli* isolates expressed third-generation cephalosporin resistance in 2021. The corresponding number in Sweden was 7.1% [2].

In addition to the emergence of Extended-spectrum beta-lactamase (ESBL)-producing Enterobacterales (EPE), carbapenem-resistant Enterobacterales are increasing in Europe, posing an even larger threat [3]. The widespread use of carbapenems promotes the increase of AMR among bacteria against this group of antibiotics, with comparatively low toxicity [4]. The importance of antibiotic stewardship to decrease AMR is widely accepted by the medical society and effective strategies are in high demand [5].

One feasible strategy for decreasing the rate of carbapenem-resistance is to explore the carbapenem-sparing alternatives. Several studies have examined the efficacy of β-lactam-β-lactamase inhibitors (BLBLIs) in infections with EPE, with conflicting results [6]. The potential inoculum effect, where some antibiotics are less effective against a high bacterial load, has been suggested as an explanation for poorer outcome with BLBLIs [7].

Furthermore, one randomized controlled trial, albeit criticized, has failed to prove the non-inferiority of BLBLIs compared to carbapenems, regarding 30-day mortality in bloodstream infections with EPE [8]. A recent meta-analysis only found increased mortality in patients treated with the BLBLI piperacillin/tazobactam (PTZ), compared to carbapenems, when the source of infection was other than urinary/biliary tract [9].

The main aim of this study was to test the non-inferiority of PTZ against carbapenems, primarily regarding 30-day mortality, in patients with bloodstream infection with EPE in Sweden, between 2013 and 2022.

## Methods

### Study design and setting

This retrospective, multicenter, non-inferiority, cohort study was set in the south of Sweden between 2013-05-26 and 2022-12-31 and included patients from all 9 public emergency care hospitals in the region of Skåne, with approximately 1.4 million inhabitants [10]. The starting date of the study was chosen when the current system of electronic medical records was introduced. Earlier medical records were not available for review. Data was collected between 2024-01-07 and 2024-05-01. The protocol and research question were framed before gathering of data. Patient identification and data collection were done in retrospect from registries and medical records.

### Participants

Patients with bloodstream infection (BSI) with EPE were identified from laboratory reports. If an individual had multiple episodes of EPE BSI (not including relapsed infection, as described below) during the study period, only one random episode was selected.

Patients were divided into two groups based on the first type of antibiotic they received in two consecutive doses: either PTZ or a carbapenem. The optimal doses of antimicrobials to appoint patients to a representative treatment group are unknown. We chose two doses, as the initial therapy is arguably the most crucial for severely ill patients [11].

Inclusion criteria were age ≥ 18 years, monomicrobial BSI due to EPE, and antibiotic treatment with PTZ or a carbapenem with minimum two dosages.

Exclusion criteria included previous administration of effective antibiotics other than PTZ/carbapenems for the same BSI (Pneumocystis prophylaxis was allowed), unavailable or missing parts of medical records, administration of PTZ/carbapenems within 48 h prior to the time of inclusion (i.e. baseline, defined as the time when blood cultures were drawn), palliative care at baseline, more than one switch between PTZ and a carbapenem and carbapenem-resistant EPE.

### Variables and definitions

The primary outcome was 30-day all-cause mortality, measured from when the positive blood cultures were taken (baseline). Secondary outcomes were ICU admission and early clinical response. Early clinical response was defined as normal vital signs on day 4 after the initiation of effective therapy: heart rate ≤ 100 bpm, temperature ≤ 37.8℃, respiratory rate ≤ 24/min, systolic blood pressure ≥ 90 mmHg, oxygen saturation ≥ 90% without supplemental oxygen [12]. In cases where vital sign data were missing, the absence of values was interpreted as normal. For an early clinical response to be achieved, all parameters were required to be within normal ranges. Additional secondary outcomes were superinfection (candidiasis and/or *Clostridioides difficile* infection during the hospital stay), relapsed infection (defined as a second positive blood culture with the same organism and phenotypic resistance as in the original blood culture, within 30 days of baseline but after a positive clinical response) and one-year mortality from baseline (the time of death after discharge was found through the population register).

The clinical frailty scale was assessed by the reviewers (TSM, AA, LM) and was discussed in the event of uncertainty [13]. The updated and revised Charlson comorbidity index was used [14]. The Pitt bacteremia score assessed the severity of illness, where a score of ≥ 4 has been associated with increased mortality [15]. Sepsis and septic shock were defined according to Sepsis-3 definitions [16]. Hospital-acquired infection was defined as a positive blood culture being drawn ≥ 48 h after hospital admission, or ≤ 48 h after previous hospital discharge [17]. The time to positive blood cultures (the shortest time in case of growth in multiple bottles) was used as a surrogate marker for bacterial load [18, 19]. The source of infection was defined by the reviewers (TSM and AA) of the medical records, largely based on culture findings, radiological examinations and the assessment of the treating physician. Source control was only applicable for conditions where surgical intervention was possible (abscess, empyema, pyelonephritis with hydronephrosis, cholangitis with choledocholithiasis, etc.) [20]. Phenotypic resistance was defined as ESBL_A_ (classical, functional class 2be β-lactamases) or ESBL_M_ (plasmid-mediated AmpC and OXA-ESBLs) [21].

### Microbiology

Microbiological data was provided by Clinical microbiology (Laboratory Medicine Skåne, Lund, Sweden), which covers all hospitals and outpatient clinics in the region. Susceptibility testing was performed and interpreted according to the EUCAST (European Committee on Antimicrobial Susceptibility Testing) methodology and breakpoints. Minimum inhibitory concentration levels were not available for most of the blood cultures. Species identification was done using matrix-assisted laser desorption/ionization time-of-flight mass spectrometry (MALDI-TOF MS: Bruker Daltonics, using the Bruker MBT Compass library version most recent at the time of sample analysis). Until December 2014, the blood culture system in use was Bactec FX (Beckton Dickinson), with BACTEC FX (BectonDickinson, Franklin Lakes, NJ) being used during the remainder of the study period. The NordicAST flowchart for when to screen for ESBL production in Enterobacterales was in use during the study period. Disk diffusion with cefpodoxime, clavulanic acid and cloxacillin was the main method used to determine the type of ESBL in cephalosporin-resistant strains.

### Study size

The largest observational, multinational, cohort study available was used for calculating the sample size [22]. The 30-day mortality was 9.8% and 13.9% for BLBLIs and carbapenems, respectively, in patients where the therapy decision was made after the susceptibility profile was identified. We chose a delta limit of 5% and 90% power with a one-sided significance level of 2.5%, which generated a total target study population of 530 individuals. A total of 644 patients were included in this study.

### Statistical methods

The Mann-Whitney U-test was chosen for continuous variables and the χ2-test or Fisher’s exact test for categorical variables. A *p* < 0.05 was considered statistically significant for most statistical analyses, except for the primary and secondary analyses, where a threshold of *p* < 0.025 was used.

Univariate logistic regression was used to identify variables associated with the primary outcome. A multivariate logistic regression analysis was also performed.

The decision to categorize patients based on their initial therapy resembles the intention-to-treat analysis commonly used in randomized controlled trials. Three cohorts were included in the primary analysis. One cohort included all study participants and two were constructed using propensity score (PS) matching. This method was chosen to adjust for confounders and estimate the effect of treatment on 30-day mortality. In the first (empirical) PS matched cohort, patients were categorized according to the first therapy they received, which involved two consecutive doses of the same antimicrobial (PTZ or carbapenem). In the second (effective) PS matched cohort, patients were categorized according to the first effective therapy (according to EUCAST breakpoints) they received, which involved two consecutive doses of the same antimicrobial.

The PS was estimated using logistic regression based on variables expected to be related to the outcome, while accounting for multicollinearity: age, gender, clinical frailty scale, Charlson comorbidity score, Pitt bacteremia score, source of infection, the pathogen in the primary blood culture [23]. One-to-one nearest matching was used, with a caliper width equal to 0.2 of the standard deviation of the logit of the PS, as is common practice [24, 25].

The standardized mean difference was < 0.1 for all matched patients, indicating a good balance (Supplementary Figs. 1 and 2) [25].

The Wald method was used to determine a 1-sided 97.5% confidence interval (CI) for risk differences [26]. To establish non-inferiority, the upper bound of the CI for the absolute risk difference in 30-day mortality, with carbapenem as the reference group, could not exceed the predefined delta limit of 5%. A sensitivity analysis with a delta limit of 2% was also conducted.

Missing data was not imputed. Complete case analysis was performed, as is common research practice [27].

Kaplan-Meier curves and log-rank tests were computed to visualize differences in one-year mortality between the treatment groups in the cohort containing all patients.

All statistical analyses were performed using RStudio software (version 2024.4.1.748) [28].

## Results

### Patient inclusion and characteristics

**Fig. 1:**
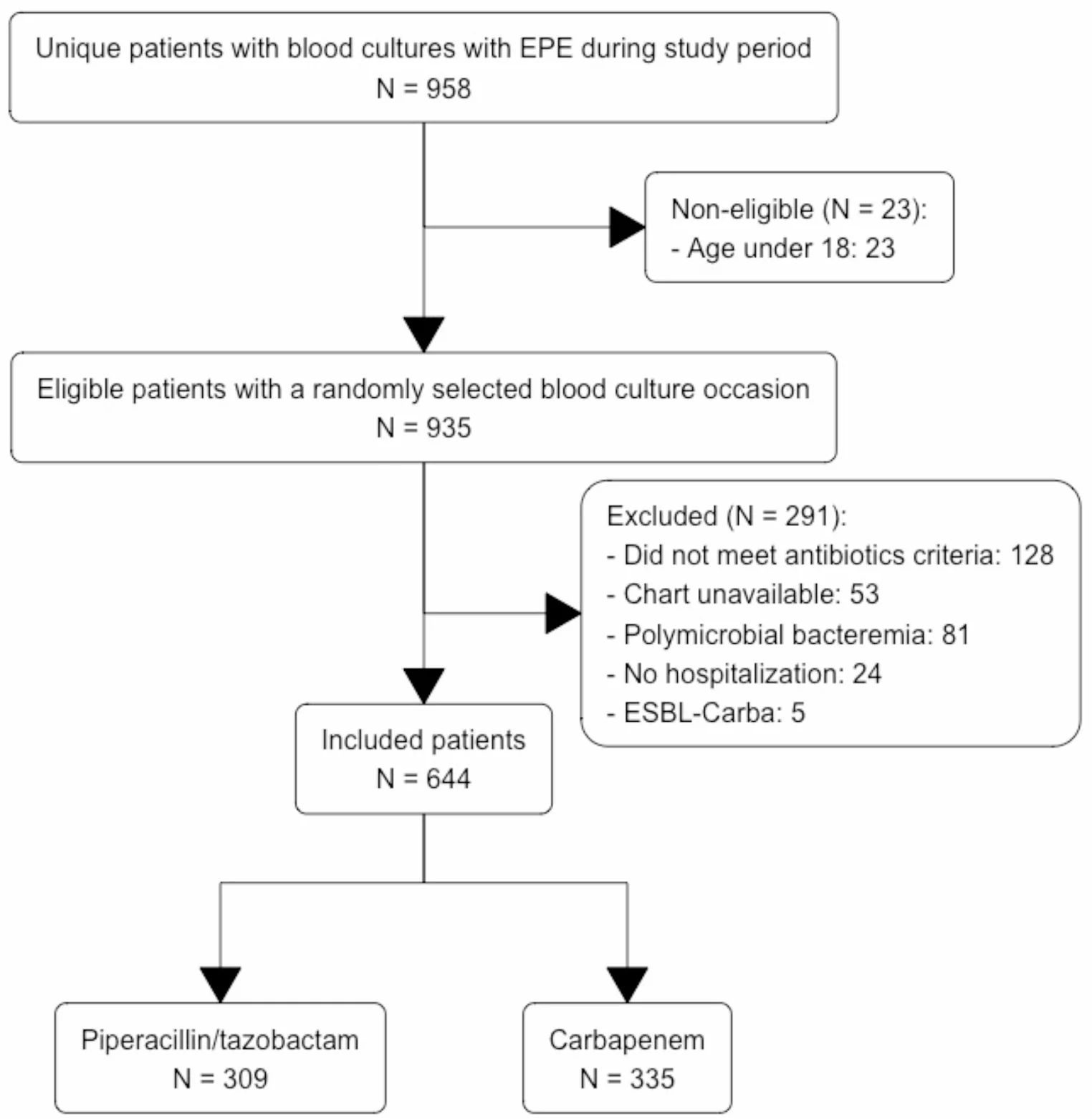
Patient inclusion and exclusion flowchart

A total of 958 unique patients with blood cultures with EPE were identified during the study period. Medical records and microbiological data of 935 adult patients were reviewed, of which 644 were included in the study after applying the exclusion criteria (Fig. 1).

Flowchart illustrating the patient inclusion process.

Both treatment groups had similar median age, gender distribution, clinical frailty scale and Charlson comorbidity score. A larger share of the patients in the PTZ group had no ceiling of treatment (Table 1).

**Table 1:** Baseline characteristics by treatment group

Variable	Carbapenem, *N* = 335^a^	PTZ, *N* = 309^a^
**Age (years)**	72 [60, 80]	73 [60, 81]
**Gender**		
Male	184 (55)	167 (54)
Female	151 (45)	142 (46)
**Ceiling of treatment** ^**b**^		
Maximum effort	242 (72)	252 (82)
No CPR	36 (11)	20 (6.5)
No CPR or intensive care	57 (17)	37 (12)
**Penicillin allergy** ^**c**^	27 (8.7)	3 (1.1)
**Clinical frailty scale**	4 [3, 7]	4 [3, 6]
**Connective tissue disease**	23 (6.9)	30 (9.7)
**Moderate or severe renal disease**	34 (10)	23 (7.4)
**Diabetes with complications**	45 (13)	28 (9.1)
**Chronic pulmonary disease**	45 (13)	51 (17)
**Congestive heart failure**	135 (40)	102 (33)
**Chronic cognitive deficit**	27 (8.1)	25 (8.1)
**Liver disease**	10 (3.0)	16 (5.2)
**Hemiplegia or paraplegia**	37 (11)	17 (5.5)
**Malignancy**	88 (26)	72 (23)
**Charlson comorbidity score** ^**d**^	2 [1, 4]	2 [0, 4]
**Psychiatric diagnosis** ^**e**^	38 (11)	42 (14)
**ESBL found in previous samples** ^**f**^	163 (49)	109 (35)

CPR: Cardiopulmonary resuscitation

^a^Median [IQR]; *N* (%)

^b^Based on medical records from before the observed hospital stay

^c^Known or suspected allergy according to medical records

^d^Determined by the reviewer of the medical records

^e^No cases of AIDS were registered

^f^Warranting daily medication

### Similar severeness of illness for both groups

Patients being treated in a tertiary hospital were more prone to receive a carbapenem compared to PTZ. The severeness of illness did not differ between the groups (Table 2).

**Table 2:** Clinical data during the hospital stay, by treatment group

Variable	Carbapenem, *N* = 335^a^	PTZ, *N* = 309^a^	*p* ^b^
**Hospitals** ^**c**^			
Tertiary	206 (61.5)	153 (49.5)	
Secondary	84 (25)	112 (36.3)	
Primary	45 (13.5)	44 (14.2)	
**Pitt bacteremia score** ^**d**^			0.75
< 4	307 (92)	281 (91)	
≥ 4	28 (8.4)	28 (9.1)	
**SOFA score** ^**e**^	3 [1, 5]	2 [2, 4]	0.34
**Sepsis** ^**f**^	211 (63)	190 (61)	0.70
**Septic shock** ^**f**^	15 (4.5)	15 (4.9)	0.81
**Lactate > 2 (mmol/L)** ^**g**^			0.28
Yes	105 (31)	115 (37)	
No	138 (41)	114 (37)	
Missing	92 (27)	80 (26)	
**Time to positive blood culture (hours)**	11 [9, 13]	11 [9, 12]	0.61
**Hospital-acquired infection**	56 (17)	37 (12)	0.087
**Source of infection**			**< 0.001**
Urinary tract	254 (76)	226 (73)	
Intra-abdominal	24 (7.2)	51 (17)	
Pneumonia	8 (2.4)	3 (1.0)	
Skin/soft-tissue	5 (1.5)	5 (1.6)	
Unknown	44 (13)	24 (7.8)	
**Source control**			0.19
Yes	31 (9.3)	33 (11)	
No	8 (2.4)	15 (4.9)	
Not applicable	296 (88)	261 (84)	
**Time to source control (hours)** ^**h**^	24 [11, 68]	29 [16, 71]	0.29
**Length of hospital stay (days)**	9 [6, 14]	8 [5, 13]	**0.023**
**Early clinical response** ^**i**^	248 (74)	225 (73)	0.78
**30-day mortality**	27 (8.1)	26 (8.4)	0.87
**One-year mortality**	92 (27)	68 (22)	0.11
**ICU admission**	21 (6.3)	16 (5.2)	0.56
**Length of ICU stay (days)**	3 [2, 5]	2 [1, 5]	0.34
**Relapsed BSI** ^**j**^			
Carbapenem previously and relapse after the current BSI	2 (0.6)	0 (0)	
PTZ previously and relapse after the current BSI	0 (0)	1 (0.3)	
Relapse after current BSI	12 (3.6)	10 (3.2)	
Carbapenem previous BSI	12 (3.6)	1 (0.3)	
PTZ previous BSI	3 (0.9)	5 (1.6)	
No	306 (91)	292 (94)	

ICU: Intensive care unit

^a^*N* (%); Median [IQR]

^b^Pearson’s Chi-squared test; Mann-Whitney U-test; Fisher’s exact test

^c^Tertiary; secondary; primary public hospitals of Skåne: Lund, Malmö; Kristianstad, Helsingborg; Trelleborg, Ängelholm, Landskrona, Hässleholm, Ystad

^d^Graded within 48 h before or on the day of the first positive blood culture. The highest point score during that time was recorded

^e^Absolute score, including habitual conditions

^f^According to Sepsis-3 definitions

^g^Within the 24 h with the highest SOFA score

^h^Only including patients with conditions where source control was possible

^i^Normal vital signs on day 4: heart rate ≤ 100 bpm, temperature ≤ 37.8℃, respiratory rate ≤ 24/min, systolic blood pressure ≥ 90 mmHg, oxygen saturation ≥ 90% without supplemental oxygen and habitual mental status

^j^All categories were defined in relation to the observed hospital stay. The first two categories had a previous and an ensuing BSI

Data on microbiology and antimicrobial therapy are provided in Supplementary Table 1.

### Treatment group was not a predictor of 30-day mortality

A total of 27 (8.1%) patients died within 30 days of baseline in the carbapenem group and 26 (8.4%) in the PTZ group, respectively (Table 2). Treatment group was not a significant predictor of 30-day mortality in a univariate analysis. Source control had a high OR, despite intervention only being applicable for a small number of patients. (Supplementary Table 2)

A multivariate logistic regression analysis was also performed (Supplementary Table 3).

### Primary outcome for the three cohorts

The absolute risk difference between the PTZ and carbapenem group was 0.3% ([1-sided 97.5% CI]: -∞ to 4.6, *p* = 0.016) indicating non-inferiority within a predefined delta limit of 5%. Statistical significance was achieved with *p* < 0.025, but this was not the case for a delta limit of 2%. Two additional PS matched cohorts were constructed: the empirical therapy and effective therapy cohorts; the groups were well balanced, as can be seen in Supplementary Figs. 1 and 2. Non-inferiority was similarly demonstrated for both cohorts, -0.4% ([1-sided 97.5% CI]: -∞ to 4.0, *p* = 0.008) and − 2.5% ([1-sided 97.5% CI]: -∞ to 1.8, *p* < 0.001), respectively, with a delta limit of 5%. After applying a stricter delta limit of 2%, non-inferiority could only be seen in the effective therapy cohort (Table 3).

**Table 3:** Primary outcome, 30-day mortality, with absolute risk difference. Delta limit set at 5% and 2% (sensitivity analysis)

Cohorts	30-day mortality PTZ, No./Total No. (%)	30-day mortality carbapenem, No./Total No. (%)	Absolute risk difference (1-sided 97.5% CI)	*p* for non-inferiority (delta limit 5%)	*p* for non-inferiority (delta limit 2%)
All patients	26/309 (8.4)	27/335 (8.1)	0.3% (-∞ to 4.6)	**0.016**	0.224
Empirical therapy	20/277 (7.2)	21/277 (7.6)	-0.4% (-∞ to 4.0)	**0.008**	0.144
Effective therapy	16/273 (5.9)	23/273 (8.4)	-2.5% (-∞ to 1.8)	**< 0.001**	**0.019**

Results are also described visually as forest plots (Supplementary Figs. 3 and 4).

### Secondary outcomes for the empirical therapy cohort

PTZ was non-inferior to carbapenems for all the secondary outcomes (the upper limit of CI was below 5%), except for the early clinical response (Table 4).

**Table 4:** Secondary outcomes for the PS matched empirical therapy cohort, with the delta limit 5%

Secondary outcomes	Cases PTZ group, No./Total No. (%)	Cases carbapenem group, No./Total No. (%)	Absolute risk difference (1-sided 97.5% CI)	*p* for non-inferiority
ICU admission	15/277 (5.4)	19/277 (6.9)	-1.5% (-∞ to 2.6)	**< 0.001**
Lack of early clinical response	71/277 (25.6)	69/277 (24.9)	0.7% (-∞ to 8.0)	0.123
Superinfection^a^	14/277 (5.1)	22/277 (7.9)	-2.8% (-∞ to 1.2)	**< 0.001**
Relapsed infection^b^	12/277 (4.3)	19/277 (6.9)	-2.6% (-∞ to 1.3)	**< 0.001**
One-year mortality	55/277 (19.9)	70/277 (25.3)	-5.4% (-∞ to 1.5)	**0.002**

^a^Candidiasis and/or *Clostridioides difficile* infection during the hospital stay

^b^Relapse before and/or after the current BSI, with the same antimicrobial therapy as for the current BSI

### Survival did not differ between the groups after one year

A crude analysis of the survival status, of all included patients, one year after baseline was assessed using Kaplan-Meier curves (Supplementary Fig. 5). Log-rank test found no statistically significant difference (*p* = 0.13) between the survival curves of the two treatment groups.

## Discussion

The findings of this retrospective, multicenter, non-inferiority, cohort study suggest that PTZ is non-inferior to carbapenems regarding 30-day mortality, for treating EPE BSI. Non-inferiority was demonstrated for all secondary outcomes, except for early clinical response. This finding could be explained by the potential inoculum effect; intra-abdominal infections, which were more frequent in the PTZ group, have been associated with higher inoculum [29]. Similarly, borderline minimum inhibitory concentrations (MICs) might be a part of the explanation; the efficacy of PTZ likely correlates to the variances in MICs within the same susceptibility category [7, 30].

The lack of non-inferiority for this outcome might influence clinicians’ choice of therapy. However, opting for carbapenems increases the risk of future AMR, highlighting a critical trade-off in clinical decision-making.

Our findings counter those of the only existing randomized controlled trial on the subject, the MERINO-trial [8]. Our treatment groups were more similar concerning baseline characteristics (including immunodeficiency), source of infection and time to effective antibiotics, compared to those of the MERINO-trial.

The time to positive blood cultures, as a proxy for bacterial load, was the same for both groups in our study. The results indicate that a higher bacterial load increases the risk of mortality, as previous studies have found [29]. A multivariate logistic regression analysis did not find a statistically significant correlation between time to positivity and the primary outcome (Supplementary Table S2).

We investigated whether source control was achieved or not (which is an established predictor of mortality) as well as the actual time to intervention, when applicable [20]. Although without statistical difference in our study, on account of the small sample size, the effect measure was substantial.

The overall mortality rate was relatively low, despite including patients dying within 96 h of the initial positive blood culture (unlike the MERINO-trial). Previous studies have seen an increased risk of mortality in patients without shock, after 3–5 h, without effective antibiotics [17]. We found a decrease in 30-day mortality by 1% for each hour to effective antibiotics (not statistically significant). This association might be explained by the inclusion of patients with shock, who received antibiotics shortly after baseline, but still had higher mortality. This study emphasized the importance of early effective treatment and attempted to replicate a realistic clinical setting, where AMR is rarely known in advance.

No non-inferiority study of this magnitude, evaluating PTZ and carbapenems in cases of EPE BSI, had previously been performed. Patients from multiple centers were included in the study, making the results applicable to a broader population. For PTZ to be considered a viable empirical alternative for treating EPE BSI, it is essential to account for the susceptibility rates among EPE in that specific region. Regions with higher prevalence of carbapenem-resistant Enterobacterales might possibly have higher prevalences of PTZ-resistant strains among their EPE [31]. Thus, decreasing the probability of PTZ being an effective empirical alternative in those regions. However, the findings of this specific study do not contradict the use of PTZ for treating EPE BSI in regions with higher AMR prevalence.

### Limitations

The retrospective study design has its limitations, such as unknown confounders and the lack of randomization. Some patients in the PTZ group had PTZ resistant bacterial strains, resulting in an ineffective initial therapy. This was never the case for patients in the carbapenem group. The choice of antimicrobial therapy might have differed between hospitals due to local guidelines or physician preference. However, there was no statistically significant difference in severity of illness (SOFA score, Pitt bacteremia score) between the treatment groups.

Although a prospective study design would have been preferable, the relatively low incidence of EPE BSI in Sweden would make for a time and resource consuming project [2]. PS matched cohorts were created to reduce confounding bias.

Data was extracted from medical records kept by different healthcare professionals, which might have caused information bias. However, no structural differences in documentation between the treatment groups were suspected.

Data concerning lab tests and vital signs were not missing completely at random. For instance, missing values of lactate were more common among patients with nosocomial BSI. Complete case analysis was performed, which introduces some selection bias and reduces the sample size. Missing vital signs were interpreted as normal values and could potentially have influenced the early clinical response outcome. The frequency of vital sign measurement might have impacted the observed outcome more than the actual effect of the chosen therapy.

Complicated patient cases were reviewed and discussed by the reviewers, to reduce potential assessment and information bias.

Non-inferiority is dependent on the chosen delta limit, as our sensitivity analysis demonstrated. The choice of delta limit was influenced by the benefit-to-risk profile of PTZ in EPE BSI. The long-term perspective favours consistently low levels of AMR to spare future lives. The delta limit for the primary analysis in this study was chosen to reproduce that of the MERINO-trial. A stricter, more clinically acceptable, delta limit was also applied to test the robustness of the findings.

Some patients experienced multiple episodes of EPE BSI during the study period. To minimize selection bias, a randomized BSI episode was selected for each patient. This approach was chosen over including only the first BSI, as this would systematically exclude subsequent episodes, potentially leading to an underestimation of the mortality risk associated with recurrent infections. Only choosing the last BSI episode would cause survival bias, where patients dying from their first BSI episode would consequently be compared to patients surviving multiple episodes. Random selection reduces the risk of overrepresentation of specific outcomes and mitigates potential correlation effects, thereby improving the robustness and generalizability of the study findings.

## Conclusion

Our findings suggest that PTZ is non-inferior to carbapenems in the treatment of bloodstream infections caused by EPE, with an acceptable 5% increase in the risk of 30-day mortality. Additionally, the results were statistically significant, with an acceptable 2% increased risk, in a PS matched cohort. PTZ may be considered an appropriate empirical therapy for patients with known or suspected EPE BSI. Future randomized controlled trials in regions with low incidence of carbapenem-resistant Enterobacterales are recommended.

## Electronic supplementary material

Below is the link to the electronic supplementary material.

Supplementary Material 1

## Data Availability

The data that support the findings of this study are not openly available due to reasons of sensitivity and are available from the corresponding author upon reasonable request. Data are located in a controlled access data storage at Lund University.
