# Supplementary material for: Piperacillin/tazobactam versus carbapenems for 30-day mortality in patients with ESBL-producing Enterobacterales bloodstream infections: a retrospective, multicenter, non-inferiority, cohort study
Source: Infection. 2025 Apr 16;53(5):1769–77. doi: 10.1007/s15010-025-02496-x (PMC12460541; doi:10.1007/s15010-025-02496-x)
Supplement: Supplementary file 1 — Supplementary Material 1 [file 15010_2025_2496_MOESM1_ESM.docx]

**Title: Piperacillin/tazobactam versus carbapenems for 30-day mortality in patients with ESBL-producing Enterobacterales bloodstream infections: a retrospective, multicenter, non-inferiority, cohort study**

**Journal: Infection**

Authors:

Thomas Sahlström Månsson^1, 5^, Alice Askemyr^4^, Torgny Sunnerhagen^3, 4^, Johan Tham^2, 5^, Kristian Riesbeck^3, 5^, Lisa Mellhammar^2, 4^

Corresponding author: Thomas Sahlström Månsson

Phone number: +46 734 33 83 72

e-mail: [Thomas.sahlstrom-mansson@med.lu.se](mailto:Thomas.sahlstrom-mansson@med.lu.se)

Postal address: Clinical Research Centre, CRC, plan 11,

Jan Waldenströms gata 35, 205 02 Malmö, Sweden

Orcid: 0000-0003-4696-4588

*^1^Department of Infectious Diseases, Sahlgrenska University Hospital, Gothenburg, Sweden.*

*^2^Department of Infectious Diseases, Skåne University Hospital, Malmö/Lund, Sweden.*

*^3^Clinical Microbiology, Infection Prevention and Control, Lund, Sweden.*

*^4^Department of Clinical Sciences Lund, division of Infection Medicine, Lund University, Lund, Sweden.*

*^5^Infectious Diseases and Clinical Microbiology, Department of Translational Medicine, Faculty of Medicine, Lund University, Malmö, Sweden.*

**Supplementary material**

Supplementary Fig. 1 Standardized mean differences (SMD) for the propensity score (PS) matched empirical cohort

Supplementary Fig. 2 Distribution of propensity scores

Supplementary Table 1 Microbiology and antimicrobial therapy, by treatment group

Supplementary Table 2 Predictors of 30-day mortality, after univariate logistic regression analysis

Supplementary Table 3 Multivariate logistic regression analysis with the outcome 30-day mortality

Supplementary Fig. 3 Absolute risk difference and non-inferiority for the three cohorts, expressed as a forest plot

Supplementary Fig. 4 The estimated crude mortality for the background population for each treatment group, expressed as a forest plot

Supplementary Fig. 5 Kaplan-Meier curves showing survival probability between baseline and one year for both treatment groups

**Supplementary Fig. 1 Standardized mean differences (SMD) for the propensity score (PS) matched empirical cohort**

**
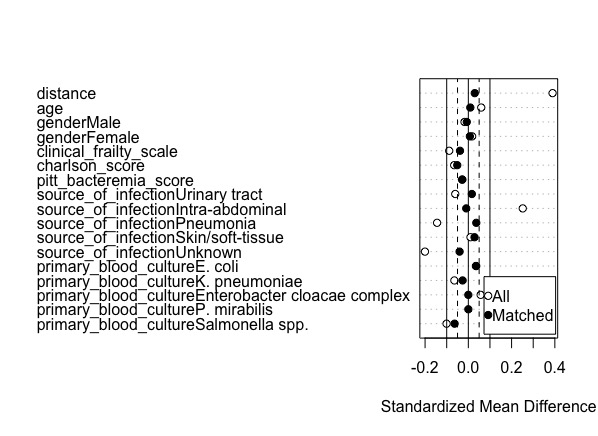
**

The SMD for each covariate before and after the PS matching process in the empirical therapy cohort. A caliper width of 0.2 standard deviations of the logit of the PS, along with the nearest-neighbor matching method, were used. Following matching, the SMD for each covariate should approach zero, indicating that the balance between the treatment groups (PTZ and carbapenem) has been successfully achieved.

**Supplementary Fig. 2 Distribution of propensity scores**


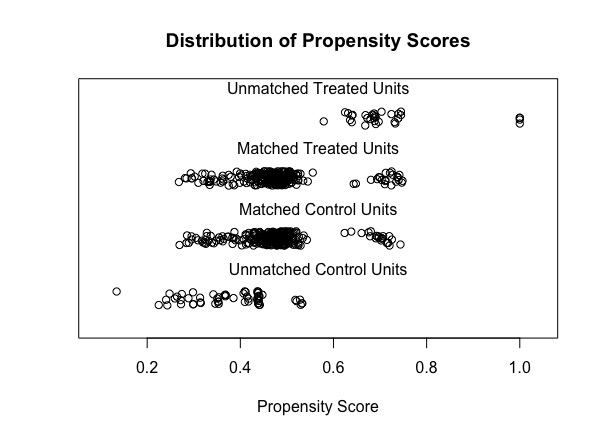


A jitter plot displaying the individual observations of each covariate in both the treatment (PTZ) and control (carbapenem) groups after matching. This plot visually represents the spread and overlap of propensity scores between the two groups, providing insight into the balance achieved through the matching process.

**Supplementary Table 1 Microbiology and antimicrobial therapy, by treatment group**

| **Variable** | **Carbapenem**, *N* = 335^a^ | **PTZ**, *N* = 309^a^ | ***p***^b^ |
| --- | --- | --- | --- |
| **Primary blood culture^c^** |  |  |  |
| *Escherichia coli* | 299 (89) | 279 (90) |  |
| *Klebsiella pneumoniae* | 33 (9.9) | 25 (8.1) |  |
| *Enterobacter cloacae* complex | 0 (0) | 1 (0.3) |  |
| *Proteus mirabilis* | 0 (0) | 3 (1.0) |  |
| *Salmonella* spp*.* | 3 (0.9) | 1 (0.3) |  |
| **Phenotypic resistance** |  |  |  |
| ESBL_A_ | 303 (90) | 283 (92) |  |
| ESBL_M_ | 26 (7.8) | 22 (7.1) |  |
| ESBL_A_ and ESBL_M_, or unknown | 6 (1.8) | 4 (1.3) |  |
| **BLBLI resistance (EUCAST SIR interpretation)** |  |  |  |
| S | 240 (73) | 259 (84) |  |
| I | 28 (8.5) | 15 (4.9) |  |
| R | 63 (19) | 33 (11) |  |
| **Time to positive blood culture (hours)** | 11 [9, 13] | 11 [9, 12] | 0.61 |
| ***Clostridioides difficile*** | 8 (2.4) | 7 (2.3) | 0.92 |
| **Candidemia** | 1 (0.3) | 0 (0) | >0.99 |
| **Oral candidiasis or candida in other culture than blood** | 19 (5.7) | 10 (3.2) | 0.14 |
| **Time to effective antibiotics (hours)** | 21 [1, 38] | 15 [1, 43] | 0.73 |
| **Days with PTZ** | 0 [0, 0] | 4 [2, 6] | **<0.001** |
| **Days with carbapenem** | 8 [4, 11] | 0 [0, 7] | **<0.001** |
| **Total effective IV treatment (days)** | 8 [5, 11] | 6 [4, 10] | **<0.001** |
| **Total effective oral treatment (days)** | 0 [0, 7] | 5 [0, 8] | **0.006** |
| **Total effective antibiotics treatment (days)** | 11 [10, 14] | 11 [9, 14] | 0.92 |
| **PTZ to carbapenem** | 0 (0) | 99 (32) | **<0.001** |
| **Escalation due to suspected therapy failure** | 0 (0) | 17 (17) |  |
| **Escalation due to antimicrobial resistance** | 0 (0) | 36 (34) |  |
| ^a^*N* (%); Median [IQR] | | | |
| ^b^Fisher's exact test; Pearson's Chi-squared test; Wilcoxon rank sum test  ^c^These were the only identified samples of EPE in this study  **Supplementary Table 2 Predictors of 30-day mortality, after univariate logistic regression analysis**   \| **Variable** \| **OR**^a^ \| **95% CI**^a^ \| ***p*** \| \| --- \| --- \| --- \| --- \| \| **Age (years)** \| 1.03 \| 1.01, 1.06 \| **0.002** \| \| **Gender** \|  \|  \| 0.16 \| \| Male \| — \| — \|  \| \| Female \| 1.50 \| 0.85, 2.65 \|  \| \| **Charlson comorbidity index** \| 1.32 \| 1.17, 1.49 \| **<0.001** \| \| **Pitt bacteremia score** \|  \|  \| **0.001** \| \| <4 \| — \| — \|  \| \| ≥4 \| 3.64 \| 1.72, 7.25 \|  \| \| **Lactate >2 (mmol/L)** \|  \|  \| **0.001** \| \| Yes \| — \| — \|  \| \| No \| 0.30 \| 0.13, 0.64 \|  \| \| Missing \| 1.07 \| 0.57, 2.02 \|  \| \| **Primary blood culture** \|  \|  \| 0.64 \| \| *Escherichia coli* \| — \| — \|  \| \| *Klebsiella pneumoniae* \| 0.82 \| 0.24, 2.11 \|  \| \| *Enterobacter cloacae* complex \| 0.00 \|  \|  \| \| *Proteus mirabilis* \| 5.52 \| 0.25, 58.6 \|  \| \| *Salmonella* spp. \| 0.00 \|  \|  \| \| **Phenotypic resistance** \|  \|  \| 0.44 \| \| ESBL_A_ \| — \| — \|  \| \| ESBL_M_ \| 0.75 \| 0.18, 2.15 \|  \| \| ESBL_A_ and ESBL_M_, or unknown \| 2.80 \| 0.42, 11.6 \|  \| \| **BLBI resistance (EUCAST SIR interpretation)** \|  \|  \| 0.37 \| \| S \| — \| — \|  \| \| I \| 1.97 \| 0.71, 4.66 \|  \| \| R \| 1.25 \| 0.55, 2.58 \|  \| \| **Time to positivity (hours)** \| 0.95 \| 0.88, 1.00 \| 0.087 \| \| **Hospital-acquired infection** \|  \|  \| **0.006** \| \| Yes \| — \| — \|  \| \| No \| 0.39 \| 0.21, 0.75 \|  \| \| **Source of infection** \|  \|  \| 0.25 \| \| Urinary tract \| — \| — \|  \| \| Intra-abdominal \| 0.91 \| 0.30, 2.20 \|  \| \| Pneumonia \| 1.27 \| 0.07, 6.93 \|  \| \| Skin/soft-tissue \| 1.41 \| 0.08, 7.84 \|  \| \| Unknown \| 2.45 \| 1.13, 4.96 \|  \| \| **Source control** \|  \|  \| 0.17 \| \| Yes \| — \| — \|  \| \| No \| 4.65 \| 0.72, 37.2 \|  \| \| Missing \| 2.92 \| 0.88, 18.2 \|  \| \| **Time to effective antibiotics (hours)** \| 0.99 \| 0.98, 1.01 \| 0.39 \| \| **Treatment group** \|  \|  \| 0.87 \| \| Carbapenem \| — \| — \|  \| \| PTZ \| 1.05 \| 0.60, 1.84 \|  \| \| ^a^OR = Odds Ratio, CI = Confidence Interval \| \| \| \| | | | |

**Supplementary Table 3** **Multivariate logistic regression analysis with the outcome 30-day mortality**

| **Variable** | **OR**^a^ | **95% CI**^a^ | ***p*** |
| --- | --- | --- | --- |
| **Age (years)** | 1.02 | 1.00, 1.05 | 0.10 |
| **Gender** |  |  | **0.016** |
| Male | — | — |  |
| Female | 2.22 | 1.16, 4.33 |  |
| **Treatment group** |  |  | 0.65 |
| Carbapenem | — | — |  |
| PTZ | 1.16 | 0.61, 2.25 |  |
| **Charlson comorbidity index** | 1.37 | 1.19, 1.59 | **<0.001** |
| **Pitt bacteremia score** |  |  | **<0.001** |
| <4 | — | — |  |
| ≥4 | 4.74 | 2.04, 10.6 |  |
| **Hospital-acquired infection** |  |  | **0.047** |
| Yes | — | — |  |
| No | 0.43 | 0.20, 0.99 |  |
| **Time to positivity (hours)** | 0.96 | 0.88, 1.00 | 0.086 |
| ^a^OR = Odds Ratio, CI = Confidence Interval | | | |

A *p*<0.05 was considered statistically significant. Variables were chosen based on known predictors of 30-day mortality, while addressing potential multicollinearity. The relatively small number of patients meeting the primary outcome did not favor a relative risk calculation as the primary analysis.

**Supplementary Fig. 3 Absolute risk difference and non-inferiority for the three cohorts, expressed as a forest plot**


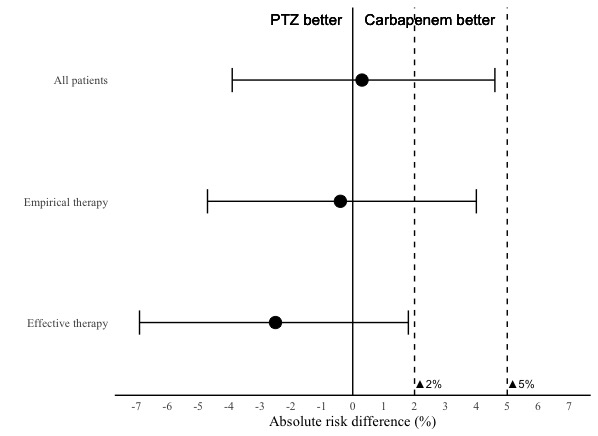


The dashed lines demonstrate the delta limits 2% and 5%. The horizontal lines represent the 95% CIs and the dot on each line represent the actual absolute risk difference.

**Supplementary Fig. 4 The estimated crude mortality for the background population for each treatment group, expressed as a forest plot**


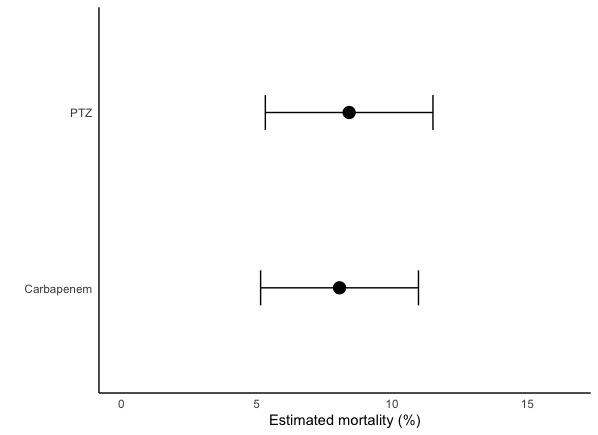


The horizontal lines represent the 95% CIs and the dot on each line represent the estimated mortality for each treatment group.

**Supplementary Fig. 5 Kaplan-Meier curves showing survival probability between baseline and one year for both treatment groups**

**
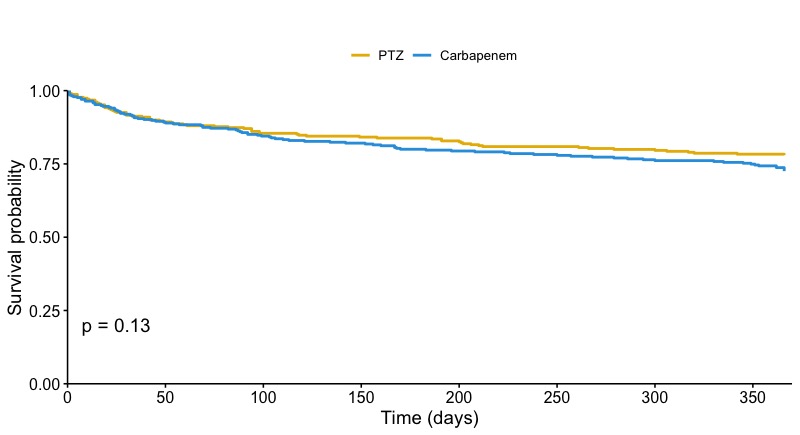
**
